# Global functional connectivity reorganization reflects cognitive processing speed deficits and fatigue in multiple sclerosis

**DOI:** 10.1111/ene.16421

**Published:** 2024-07-26

**Authors:** Pavel Hok, Quang Thong Thai, Barbora Rehák Bučková, Martin Domin, Kamila Řasová, Jaroslav Tintěra, Martin Lotze, Matthias Grothe, Jaroslav Hlinka

**Affiliations:** ^1^ Department of Neurology University Medicine Greifswald Greifswald Germany; ^2^ Functional Imaging Unit Institute of Diagnostic Radiology and Neuroradiology, University Medicine Greifswald Greifswald Germany; ^3^ Department of Neurology, Faculty of Medicine and Dentistry Palacký University Olomouc Olomouc Czechia; ^4^ Department of Complex Systems Institute of Computer Science of the Czech Academy of Sciences Prague Czechia; ^5^ Department of Cognitive Neuroscience Radboud University Medical Centre Nijmegen The Netherlands; ^6^ Department of Rehabilitation, Third Faculty of Medicine Charles University Prague Czechia; ^7^ Radiodiagnostic and Interventional Radiology Department Institute for Clinical and Experimental Medicine Prague Czechia

**Keywords:** biomarkers, cognitive processing speed, fatigue, fMRI, multiple sclerosis

## Abstract

**Background and purpose:**

Cognitive impairment (CI) in multiple sclerosis (MS) is associated with bidirectional changes in resting‐state centrality measures. However, practicable functional magnetic resonance imaging (fMRI) biomarkers of CI are still lacking. The aim of this study was to assess the graph‐theory‐based degree rank order disruption index (*k*_D_) and its association with cognitive processing speed as a marker of CI in patients with MS (PwMS) in a secondary cross‐sectional fMRI analysis.

**Methods:**

Differentiation between PwMS and healthy controls (HCs) using *k*_D_ and its correlation with CI (Symbol Digit Modalities Test) was compared to established imaging biomarkers (regional degree, volumetry, diffusion‐weighted imaging, lesion mapping). Additional associations were assessed for fatigue (Fatigue Scale for Motor and Cognitive Functions), gait and global disability.

**Results:**

Analysis in 56 PwMS and 58 HCs (35/27 women, median age 45.1/40.5 years) showed lower *k*_D_ in PwMS than in HCs (median −0.30/−0.06, interquartile range 0.55/0.54; *p* = 0.009, Mann–Whitney *U* test), yielding acceptable yet non‐superior differentiation (area under curve 0.64). *k*_D_ and degree in medial prefrontal cortex (MPFC) correlated with CI (*k*_D_/MPFC Spearman's *ρ* = 0.32/−0.45, *p* = 0.019/0.001, *n* = 55). *k*_D_ also explained fatigue (*ρ* = −0.34, *p* = 0.010, *n* = 56) but neither gait nor disability.

**Conclusions:**

*k*_D_ is a potential biomarker of CI and fatigue warranting further validation.

## Graphical abstract

Multiple sclerosis was associated with global disruption of degree rank ordering (*k*_D_), a graph‐theoretical measure of resting‐state functional connectivity. Degree rank order disruption explained deficits in cognitive processing speed and fatigue score, but was not correlated with gait or global disability assessments. Whilst slowing of cognitive processing was associated with increased degree centrality in the medial prefrontal cortex, fatigue was accompanied by a higher degree in the cerebellum and caudate nuclei and a lower degree in the left fusiform gyrus.

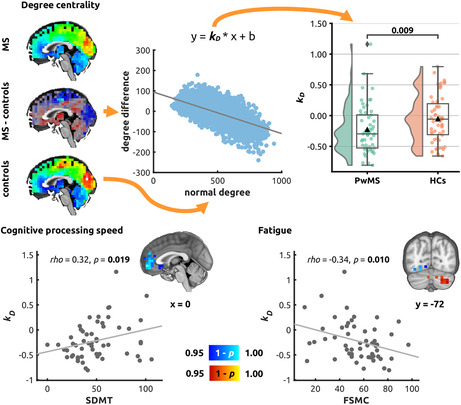

## INTRODUCTION

Multiple sclerosis (MS) is frequently associated with cognitive impairment (CI) [1]. Being often neglected in routine neurological examination [1, 2], CI and its imaging biomarkers, such as grey matter volume, white matter integrity or resting‐state functional connectivity (rsFC), are attracting increasing attention [1, 3]. Recent resting‐state functional magnetic resonance imaging (fMRI) studies utilizing graph‐theoretical approaches have identified a relationship between CI and decreased overall mean degree [4] or increased centrality in the default mode network (DMN) accompanied by decreased centrality outside the DMN [5, 6, 7, 8, 9, 10]. Moreover, changes in centrality also seem to precede the actual cognitive decline, indicating its potential utility as a prognostic biomarker [6]. However, the ultimate goal, that is, providing individual predictions applicable in day‐to‐day practice, remains far from achieved, thus urging further refinement of imaging and analytical methods [3].

Whereas a voxel‐wise centrality assessment would require considerable time and personal resources, a recently introduced centrality‐derived global scalar metric, the degree rank order disruption index (*k*_D_) [11], could serve as a biomarker reflecting simultaneous focal increases and decreases in degree centrality throughout the brain, requiring less demanding interpretation. The *k*_D_ has previously been demonstrated to be associated with brain‐wide degree centrality changes in impaired consciousness [12] and chronic pain [11], but so far it was evaluated neither as a biomarker of the MS‐related brain damage nor as a predictor of CI in MS.

Hence, the aim of this study was to investigate the relationship between *k*_D_ and the presence of MS and its clinical presentation (reduced cognitive processing speed as a marker of global CI) in comparison to established multimodal MRI biomarkers (resting‐state fMRI with regional degree assessment, diffusion‐weighted imaging [DWI], volumetry and lesion mapping) in patients with MS and matched healthy controls. The hypotheses were as follows: (1) *k*_D_ differs between patients with MS (PwMS) and matched healthy controls (HCs); (2a) *k*_D_ is superior to the regional degree centrality in the pre‐selected regions of interest (ROIs) in differentiating between PwMS and HCs and (2b) improves such differentiation when used in conjunction with established structural imaging diagnostic biomarkers of MS (lesion load, global atrophy, global white matter integrity); (3) *k*_D_ correlates with deficits in cognitive processing speed in PwMS; (4a) *k*_D_ provides superior correlation with cognitive processing speed in comparison to the regional degree centrality in pre‐selected ROIs and (4b) improves the regression model of cognitive processing speed when added on top of established structural imaging diagnostic biomarkers of MS.

In addition, the following exploratory hypotheses were tested to assess associations between *k*_D_ and potential confounding factors: (5) *k*_D_ correlates with global disability, fatigue and motor performance (gait) and (6) improves regression models for these clinical outcomes when used jointly with the established structural imaging diagnostic biomarkers of MS (lesion load, global atrophy, global white matter integrity); (7) *k*_D_ correlates with these structural imaging biomarkers; and (8) the structural imaging diagnostic biomarkers of MS differ between PwMS and matched HCs.

## METHOD

### Study design and participant selection

The secondary analysis was performed on cross‐sectional imaging and behavioural data of 65 PwMS and 65 HCs matched for age and sex, with participants recruited from MS centres across Czechia. The same cohort has been partially analysed and published using other methods [13, 14]. Original inclusion criteria were the diagnosis of MS [15]; spastic paraparesis as a prominent clinical feature; stable clinical status for at least 3 months preceding the study (determined by a neurologist); physical ability to undergo clinical testing—consistent with an Expanded Disability Status Scale (EDSS) score ≤7.5. Subjects were excluded in the case of missing imaging data or imaging artefacts and conditionally excluded if exceeding the motion outlier criteria (see the section ‘Pre‐processing’ in the Supplementary Methods). To address potential attrition bias, a sensitivity analysis was performed in a sample including motion outlier subjects.

### Clinical assessment and questionnaires

Clinical parameters comprised an objective assessment of cognitive performance using a Symbol Digit Modalities Test (SDMT) [16] and possible confounding factors such as fatigue (Fatigue Scale for Motor and Cognitive Functions, FSMC) [17], global disability (EDSS) [18] and motor performance (Timed Up and Go Test, TUG) [19].

### Standard protocol approvals, registrations and patient consents

The secondary data analysis was pre‐registered at osf.io (https://osf.io/v8ejw). The original study was approved by the Ethics Committee of the Faculty Hospital Královské Vinohrady, approval no. EK‐VP/22/0/2014. All patients gave their written informed consent to participate in the study.

### MRI data acquisition

Imaging was performed using a 3 T magnetic resonance scanner (Siemens Trio Tim, Erlangen, Germany) equipped with a 12‐channel phased‐array head coil. The MRI protocol included blood oxygenation level dependent resting‐state fMRI, as well as high‐resolution 1‐mm T1‐weighted and T2‐weighted imaging and 2‐mm DWI. Detailed acquisition parameters have been published elsewhere [13, 14].

### Imaging data analysis

Following the pre‐processing, subject‐specific functional connectivity matrices containing Fisher‐transformed Pearson's *r* coefficients were computed in CONN toolbox v. 21a [20] for 4632 large voxels created using 6‐mm resampling of a common grey matter mask (see Supplementary Methods for more details). Degree centrality was then computed for each subject using a brain connectivity toolbox (https://sites.google.com/a/brain‐connectivity‐toolbox.net/bct/) with 10% link density [11]. Regional degree centrality was extracted by averaging nodal degree from the DMN (four ROIs) [5, 6, 8], basal ganglia (six ROIs) [6, 8], thalamus, hippocampus and cerebellum (five ROIs) [8] and from the multimodal ROIs explicitly participating in the SDMT: superior parietal lobule (two ROIs), dorsolateral prefrontal cortex (two ROIs) and anterior cingulate cortex (ACC) (1 ROI) [21] (see Figure S1 and Table S1). Finally, *k*_D_ was calculated using custom MATLAB scripts implementing a modified approach according to Mansour et al. [11], as described in Supplementary Methods.

The pre‐processing of T1‐weighted and DWI data, the calculation of grey matter volume (GMV) as a measure of cortical atrophy and extraction of fractional anisotropy (FA) as a measure of white matter integrity, as well as the ROI definition for GMV and FA are described elsewhere [14]. Finally, the lesion load (LL) was calculated using the lesion segmentation tool (https://www.statistical‐modelling.de/lst.html) with the lesion prediction algorithm [22].

### Statistical analysis

Initially, normality was assessed for all continuous variables using the Kolmogorov–Smirnov test. Non‐parametric tests were applied in case normality was violated. Additionally, regressors considerably deviating from the normal distribution (LL) were log‐transformed prior to any subsequent analysis to meet regression model assumptions. Demographic variables were compared between groups using Fisher's exact test and the Mann–Whitney *U* test. Pairwise deletion was applied in the case of missing clinical data. A summary of all variables, outcome measures and statistical tests for each hypothesis is provided in Table S2. All tests were performed using SPSS v29.0.1.1 (IBM, Armonk, NY, USA). *p* < 0.05 was considered significant. For correlations of regional degree centrality (4a), Bonferroni–Holm correction for multiple comparisons across 18 ROIs was applied (*α* = 0.0028). One‐tailed tests were used where superiority was assumed by the hypotheses or statistics with one‐tailed distribution were employed (2a, 2b, 4b and 6), with two‐tailed tests applied otherwise. Details on figure preparation, power analysis as well as additional post hoc analyses are provided in Supplementary Methods.

## RESULTS

### Study sample

Out of the original sample of 65 PwMS and 65 HCs, one PwMS and one HC were excluded due to missing data (incomplete field of view and susceptibility artefact) and another PwMS was excluded due to a suspected vascular lesion. In the remaining sample, 13 subjects with excessive motion levels were identified (seven PwMS and six HCs) (see Figure S2 for the inclusion/exclusion diagram). Here, only results in 56 PwMS and 58 HCs after excluding motion outliers are reported (‘final’ sample), whereas results in the sample with outliers are provided in Supplementary Results. Whilst both analyses yielded mostly similar results, two differences are explicitly stated below. Demographic details of the ‘final’ sample and summary statistics for clinical parameters are provided in Table 1.

**TABLE 1:** Demographic and clinical data.

Variable		Statistic	PwMS	HC	*p*
Number		Count	56	58	
Sex [women/men]			35/21	27/31	0.095^a^
Age [years]		Median ± IQR	45.1 ± 17	40.5 ± 17	0.090^b^
Diagnosis	RRMS	Count, %	35, 62.5%		
	SPMS		15, 26.8%		
	PPMS		5, 8.9%		
	no data		1, 1.8%		
Time since diagnosis [years]		Mean ± SD	12.6 ± 6.2		
EDSS		Median ± IQR	4.5 ± 2.5		
SDMT			45 ± 29		
FSMC			57 ± 23		
TUG [s]			10.3 ± 9		

Abbreviations: EDSS, Expanded Disability Status Scale; FSMC, Fatigue Scale for Motor and Cognitive Functions; HCs, healthy controls; IQR, interquartile range; MS, multiple sclerosis; PPMS, primary progressive MS; PwMS, patients with MS; RRMS, relapsing–remitting MS; SD, standard deviation; SDMT, Symbol Digit Modalities Test; SPMS, secondary progressive MS; TUG, Timed Up and Go Test.

^a^ Fisher's exact test.

^b^ Mann–Whitney *U* test.

### Group differences and differentiation between PwMS and HCs

**FIGURE 1:**
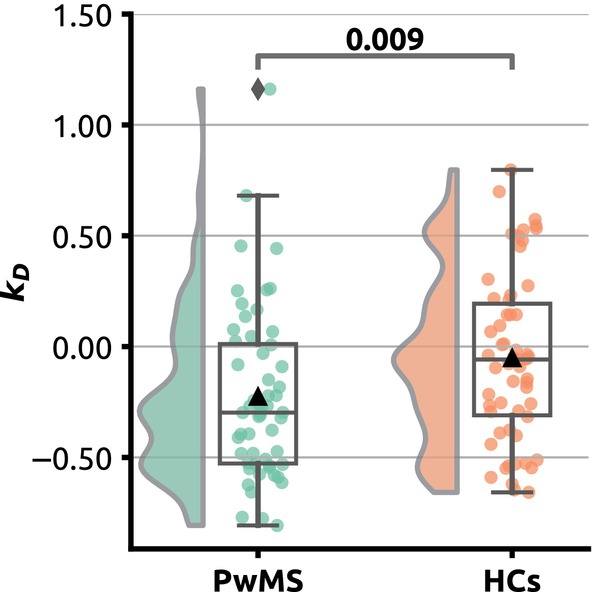
Group differences in degree rank order disruption index (*k*_D_). Raincloud plots illustrating individual *k*_D_ values and distribution. Patients with multiple sclerosis are shown in green, healthy controls in red. Mann–Whitney *U* test *p* value is provided in annotation.

Patients with MS showed significantly lower *k*_D_ compared to HCs (PwMS, median −0.298, interquartile range [IQR] 0.549; HCs, median −0.058, IQR = 0.542; *p* = 0.009; Mann–Whitney *U* test) (Figure 1). Underlying raw degree centrality data are summarized in Figure S3.

**FIGURE 2:**
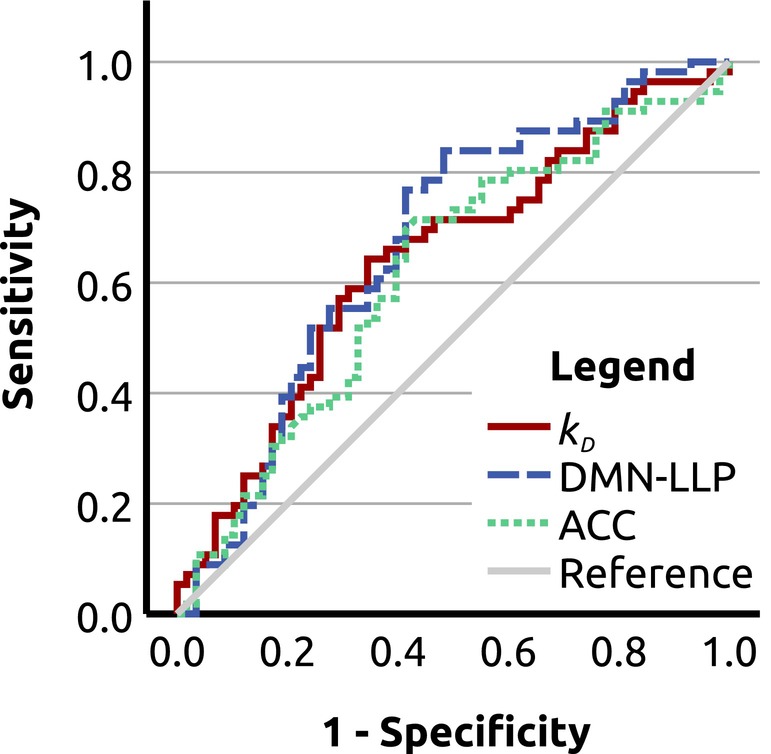
Receiver operating characteristic (ROC) analysis. ROC curves for differentiation between patients with multiple sclerosis and healthy controls using degree rank order disruption index (*k*_D_) (area under the curve [AUC] = 0.642; *p* = 0.007; two‐tailed asymptotic significance for null hypothesis AUC = 0.5, uncorrected); the left lateral parietal portion of the default mode network (DMN‐LLP; AUC = 0.671; *p* = 0.001) and the anterior cingulate cortex (ACC; AUC = 0.619; *p* = 0.026).

The ROC analysis for differentiation between PwMS and HCs yielded significant above‐chance area under curve (AUC) for *k*_D_ (AUC = 0.642, *p* = 0.007; two‐tailed asymptotic significance for null hypothesis AUC = 0.5), the left lateral parietal portion of the DMN (AUC = 0.671, *p* = 0.001) and the ACC (AUC = 0.619; *p* = 0.026) (Figure 2 and Table S3). In pairwise comparisons, AUC for *k*_D_ was significantly higher than AUC for six ROIs and did not significantly differ from the remaining ROIs (Table S3).

No significant improvement was observed in a multiple logistic regression model differentiating between PwMS and HCs after adding *k*_D_ as an additional regressor on top of GMV, FA, log(LL) (*χ* ^*2*^ step 0.007, *p* = 0.934).

### Correlation with cognitive processing speed

**FIGURE 3:**
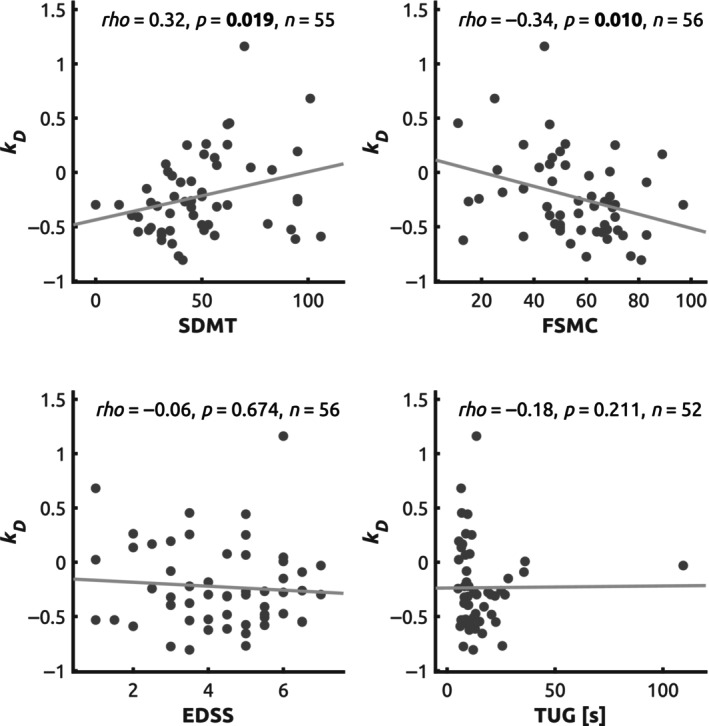
Correlation between *k*_D_ and clinical scores. Scatter plots illustrating relationship between the degree rank order disruption index (*k*_D_) and cognitive processing speed (Symbol Digit Modalities Test, SDMT), global disability (Expanded Disability Status Scale, EDSS), fatigue (Fatigue Scale for Motor and Cognitive Functions, FSMC) and motor performance (Timed Up and Go Test, TUG). Spearman's rank correlation coefficient (*rho*), two‐tailed uncorrected significance and number of valid observations are provided.

A significant correlation was detected between *k*_D_ and SDMT (Spearman's *ρ* = 0.32, *p* = 0.019, *n* = 55, Figure 3). For the regional degree centrality, significant correlation with SDMT was observed in the medial prefrontal part of the DMN, yielding slightly higher effect size than the *k*_D_ (Table 2). An ordinal regression model including GMV, FA, log(LL), age, sex and years since diagnosis as regressors of the SDMT score was significantly improved after adding *k*_D_ (*χ* ^2^ step 4.49, *p* = 0.034, likelihood ratio test; see Table S4). In contrast, neither significant correlation with SDMT nor improvement of the regression model for SDMT were observed in the analysis with motion outliers (see Supplementary Results).

**TABLE 2:** Correlation between regional degree and clinical scores.

ROI	SDMT *n* = 55		FSMC *n* = 56	
	*ρ* ^a^	*p* ^a^	*ρ* ^a^	*p* ^a^
DMN‐MPFC	**−0.45**	**0.001**	0.23	0.081
DMN‐LP				
L	−0.03	0.847	0.12	0.365
R	−0.28	0.036	0.27	0.041
DMN‐PCC	−0.18	0.177	0.13	0.346
Putamen				
L	−0.35	0.009	0.38	0.004
R	−0.27	0.044	0.30	0.024
Caudate nucleus				
L	−0.35	0.008	**0.41**	**0.002**
R	−0.38	0.004	**0.40**	**0.002**
Thalamus				
L	−0.27	0.043	0.30	0.025
R	−0.27	0.046	0.25	0.059
Hippocampus				
L	−0.24	0.084	0.23	0.085
R	−0.33	0.015	0.26	0.053
Cerebellum	−0.25	0.067	0.34	0.010
Superior parietal lobule				
L	−0.04	0.781	−0.21	0.118
R	−0.12	0.396	−0.12	0.369
DLPFC				
L	−0.05	0.728	−0.24	0.069
R	−0.11	0.424	0.01	0.913
ACC	−0.21	0.120	0.06	0.657

Abbreviations: ACC, anterior cingulate cortex; DLPFC, dorsolateral prefrontal cortex; DMN, default mode network; DMN‐LP, lateral parietal part of the DMN; DMN‐MPFC, DMN ‐ medial prefrontal cortex; DMN‐PCC, DMN ‐ posterior cingulate cortex; FSMC, Fatigue Scale for Motor and Cognitive Functions; L, left; R, right; ROI, region of interest; SDMT, Symbol Digit Modalities Test; SPL, superior parietal lobule.

^a^ Spearman's rank correlation coefficient *ρ*, significant correlations at Bonferroni–Holm‐corrected *α* = 0.0028 are in bold type.

### Correlation with fatigue, global disability and motor performance

**FIGURE 4:**
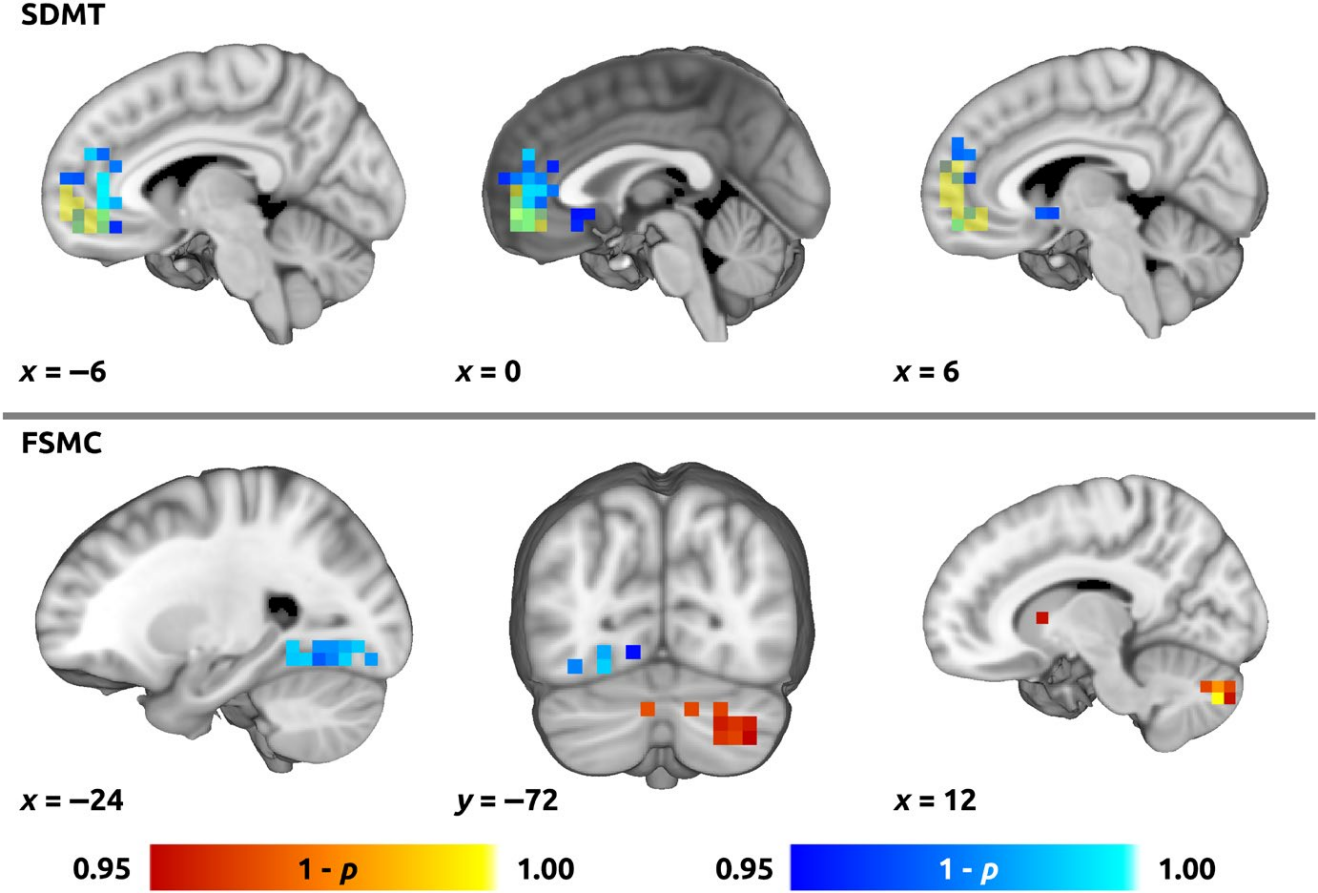
Voxel‐wise correlation between degree and clinical scores. Colour overlays illustrating spatial distribution of correlation of the voxel‐wise degree centrality with (top) cognitive processing speed (Symbol Digit Modalities Test, SDMT) and (bottom) fatigue (Fatigue Scale for Motor and Cognitive Functions, FSMC). Red–yellow and blue–light blue overlays indicate positive and negative correlation, respectively. Thresholded using non‐parametric threshold‐free cluster enhancement (10,000 permutations, family‐wise error‐corrected *p* = 0.05). In the top panel, yellow overlay shows overlap with the medial prefrontal cortex default mode network (MPFC‐DMN) region of interest.

A significant correlation was detected between *k*_D_ and FSMC (Spearman's *ρ* = −0.34, *p* = 0.010, *n* = 56) but not for EDSS (Spearman's *ρ* = −0.06, *p* = 0.674, *n* = 56) or TUG (Spearman's *ρ* = −0.18, *p* = 0.211, *n* = 52) (Figure 4). In ordinal regression, *k*_D_ significantly improved the model fit for fatigue (FSMC) when added on top of GMV, FA, log(LL), age, sex and years since diagnosis, but not for EDSS or TUG (Table S4).

### Relationship between *k*_D_ and structural imaging biomarkers

No significant correlation was observed between *k*_D_ and structural imaging parameters, that is, GMV, LL and global FA (see Figure S4). In analysis with motion outliers, however, *k*_D_ was significantly correlated with LL (see Supplementary Results). All structural imaging parameters significantly differed between PwMS and HCs (see Table S5).

### Analysis with motion outliers

For complete results of the sensitivity analysis with motion outliers, see Supplementary Results and Tables S6–S10.

### Post hoc analyses

Voxel‐wise group differences are illustrated in Figure S3. Voxel‐wise regression analysis for degree centrality as response variable and SDMT as explanatory variable yielded significant clusters with negative effect mainly in medial prefrontal cortex (MPFC) and ACC, and to a lesser degree in the subcallosal cortex and the right nucleus accumbens, overlapping in part with the DMN‐MPFC ROI (Figure 4, Figure S3 for unthresholded data). The regression for FSMC yielded a positive effect in predominantly the right cerebellum, right caudate nucleus, right inferior frontal gyrus and a negative effect in the left temporo‐occipital fusiform and lateral occipital cortex (Figure 4, Figure S3 for unthresholded data).

The ROI analysis for FSMC yielded significant correlation in the left and right caudate nuclei (left, Spearman's *ρ* = 0.41, *p* = 0.002; right, Spearman's *ρ* = 0.40, *p* = 0.002; *n* = 56; Bonferroni–Holm‐corrected across the 18 ROIs, i.e., *α* = 0.0028). See Table 2 for complete results.

## DISCUSSION

The present study aimed to investigate the potential utility of *k*_D_ as a new functional imaging biomarker of cognitive processing speed (and hence of CI) in MS in comparison to regional degree centrality. Whilst *k*_D_ in PwMS significantly differed from HCs and was a significant explanatory variable for cognitive processing speed (SDMT) in PwMS, it yielded weaker correlation than the mean degree centrality in the frontal hub (MPFC) of the DMN. In an exploratory analysis, *k*_D_ turned out to be a significant explanatory variable for self‐reported fatigue. A post hoc analysis indicated that the correlation with fatigue might be driven by degree centrality changes in the cerebellum, basal ganglia (caudate nuclei) and left fusiform gyrus. These results were shown to be largely independent of structural imaging parameters, which were not significantly correlated with *k*_D_.

### Differentiation between PwMS and HCs

Our primary observation of decreased *k*_D_ in PwMS captures the global character of changes in nodal centrality (both degree and eigenvector) that have recently been reported on the local and network‐wide level in MS [5, 7, 8, 23, 24]. Lower *k*_D_ in PwMS suggests less centralized and more diffusely distributed rsFC, which is analogous to a previously described disruption of the rich‐club topology of the brain network in MS [25]. However, our multiple regression analysis indicated that *k*_D_ did not improve differentiation between PwMS and HCs when added on top of structural imaging parameters. Taken together with the considerable overlap between *k*_D_ distributions in PwMS and HCs (Figure 1), it can be inferred that degree reordering is not primarily driven by the mere presence of MS and is more probably related to the resulting neurological deficits.

### Correlation with cognitive processing speed

Our next main analysis demonstrated that *k*_D_ was a significant explanatory variable for cognitive processing speed compared to global structural imaging parameters. Whilst the negative correlation between *k*_D_ and SDMT is a novel finding, it is in line with previous evidence for a weak positive association between SDMT score and rsFC of the peripheral nodes outside the rich club [25]. The correlation between *k*_D_ and SDMT yielded an effect size similar to some previously reported structural imaging biomarkers of cognitive processing speed, including fractional anisotropy in the superior longitudinal fascicle [26] or grey matter atrophy‐based brain age gap [27], but was lower than overall effect size in a recent meta‐analysis of multimodal structural MRI data [28].

In our dataset, a superior correlation with SDMT was achieved using regional degree centrality in the MPFC hub of the DMN, yielding an effect size comparable with structural imaging biomarkers [28, 29]. Correspondingly, cognitive impairment in MS has been shown to be associated with increased degree or eigenvector centrality in the DMN [6, 7, 8, 9, 10] and decreased centrality in the visual [7, 9, 10] and sensorimotor network [7], whilst eigenvector and degree centrality show high agreement even within the same group [8]. Nevertheless, our results add novel evidence for the strength of the relationship between degree centrality in the DMN and cognitive processing speed since no such correlation in MS has been reported before.

### Role of the DMN in pathophysiology of cognitive deterioration in MS

The ROI and post hoc voxel‐wise analyses indicated that the MPFC and ACC (i.e., anterior DMN) provided the highest correlation between degree and cognitive processing speed. The role of abnormalities in DMN in CI remains controversial, possibly being non‐specifically linked to the dysfunction of the entire brain network [3]. However, an excessively central and less dynamic DMN has also been proposed to directly hinder externally oriented cognitive processing by superfluous introspective thoughts [7]. Additionally, rsFC studies using the Paced Auditory Serial Addition Test also point to dysfunction of DMN and subcallosal cortex [24, 29]. Hence, graph‐theoretical measures extracted from the anterior portion of the DMN are potential future candidates for even more accurate biomarkers of cognitive processing speed than *k*_D_.

### Association with fatigue

Our results indicate that, cognitive processing speed aside, *k*_D_ was also a significant explanatory variable for the self‐reported global fatigue score (FSMC). Whilst cognitive performance and fatigue have been shown to be associated with similar rsFC dysfunctions, such as increased rsFC in posterior DMN and reduced rsFC in the anterior DMN [30], our post hoc analyses on ROI and voxel‐wise level suggested potential differentiation between mechanisms underlying cognitive decline and global fatigue. Whereas SDMT correlated with average degree in DMN‐MPFC, fatigue scores were more strongly associated with degree in the caudate nuclei, cerebellum and fusiform cortex (Table 2 and Figure 4).

From the network perspective, fatigue has been associated with damage to cortico‐subcortical pathways and with particular involvement of the prefrontal cortex [31]. On the computational (metacognitive) level, it has been proposed to result from mismatch between predicted and measured output from cognitive and sensorimotor networks [3, 32]. Our results fit in by emphasizing the role of basal ganglia [33] and cerebellum, which is involved in maintaining internal forward models and error monitoring [34]. Future dedicated studies should evaluate the specificity and stability of the here identified biomarkers and their accuracy with respect to the motor and cognitive sub‐domains of FSMC.

### Limitations and future directions

Whilst the main strength of the study was rigorous pre‐registration of all main analyses, there are also several limitations related to the fact that this study was carried out as a secondary analysis: SDMT reflects mainly cognitive processing speed and involves visual processing. Further assessments across multiple cognitive domains and sensory modalities as well as consideration of depression as a potential confounding factor are warranted. Furthermore, normative data, assessment of test–retest reliability, consideration of compound predictive models (involving regional degree centrality) and longitudinal evaluation are also necessary. Finally, our data cannot be currently generalized to all individuals with MS (see our inclusion criteria); hence, a cross‐validation of our results in an independent dataset is necessary before translating the results into clinical practice.

## CONCLUSION

Although our results require further cross‐validation, they suggest that obtaining a single scalar functional imaging biomarker of cognitive processing speed and CI in general is feasible and may provide an important diagnostic tool to assess performance decline due to CI and fatigue.

## AUTHOR CONTRIBUTIONS

**Pavel Hok:** Writing – original draft; writing – review and editing; conceptualization; methodology; software; formal analysis; visualization. **Quang Thong Thai:** Formal analysis; writing – review and editing. **Barbora Rehák Bučková:** Investigation; conceptualization; methodology; data curation; writing – review and editing. **Martin Domin:** Software; formal analysis; writing – review and editing; writing – original draft. **Kamila Řasová:** Investigation; writing – review and editing. **Jaroslav Tintěra:** Investigation; writing – review and editing. **Martin Lotze:** Conceptualization; writing – review and editing; supervision; resources. **Matthias Grothe:** Writing – review and editing; conceptualization; supervision. **Jaroslav Hlinka:** Conceptualization; methodology; investigation; supervision; writing – review and editing; writing – original draft; validation.

## FUNDING INFORMATION

P. Hok was awarded a Gerhard‐Domagk fellowship by University Medicine Greifswald for undertaking this study. K. Řasová was supported by Charles University, programme Cooperatio (Neuroscience) and 260648/SVV/2024. J. Hlinka was supported by the ERDF‐Project Brain dynamics, No. CZ.02.01.01/00/22_008/0004643.

## CONFLICT OF INTEREST STATEMENT

None.

## Supporting information


Data S1.

## Data Availability

Part of the dataset (imaging data for 60 PwMS with the respective global disability and motor scales) is publicly available in an on‐line repository at https://osf.io/p2kj7/. Data not provided in the article may be shared (anonymized) at the request of any qualified investigator for purposes of replicating procedures and results, upon signing a data sharing agreement. The custom MATLAB script for *k*_D_ calculation is available at https://github.com/pavelhok/calculate_kd/tree/MS‐project.
